# Predictive relevance of HOXB13 protein expression for tamoxifen benefit in breast cancer

**DOI:** 10.1186/bcr2612

**Published:** 2010-07-22

**Authors:** Piiha-Lotta Jerevall, Agneta Jansson, Tommy Fornander, Lambert Skoog, Bo Nordenskjöld, Olle Stål

**Affiliations:** 1 Department of Clinical and Experimental Medicine, Division of Oncology, Faculty of Health Sciences, Linköping University, SE-58185 Linköping, Sweden; 2 Department of Oncology, Karolinska University Hospital, Stockholm South General Hospital, Sjukhusbacken 10, SE-11883 Stockholm, Sweden; 3 Department of Clinical Pathology and Cytology, Karolinska University Hospital, Solna, SE-17176 Stockholm, Sweden

## Abstract

**Introduction:**

The *HOXB13:IL17BR *index has been identified to predict clinical outcome in the setting of adjuvant tamoxifen monotherapy of breast cancer. Further studies have shown that *HOXB13 *in particular can indicate benefit of prolonged tamoxifen treatment. Patients with high-expressing tumors did not benefit from prolonged treatment, suggesting that *HOXB13 *might be involved in tamoxifen resistance. No studies have been made regarding the HOXB13 protein levels in breast cancer. The aim of our study was to investigate whether tamoxifen benefit can be correlated to different levels of HOXB13 protein expression.

**Methods:**

We used immunohistochemistry to analyze protein levels of HOXB13 in tumor samples from 912 postmenopausal node-negative breast cancer patients randomized to adjuvant tamoxifen therapy or no endocrine treatment.

**Results:**

Tamoxifen-treated patients with estrogen receptor-positive tumors expressing none or low levels of HOXB13 had a clear benefit from tamoxifen in terms of longer distant recurrence-free survival (DRFS) (hazard ratio = 0.38, 95% confidence interval = 0.23 to 0.60, *P *= 0.000048). However, for patients with a high or intermediate HOXB13 tumor expression, tamoxifen did not prolong the DRFS compared with the untreated patients (hazard ratio = 0.88, 95% confidence interval = 0.47 to 1.65, *P *= 0.69). Interaction between HOXB13 expression and benefit from tamoxifen was statistically significant for DRFS (*P *= 0.035). No prognostic value could be ascribed to HOXB13 among systemically untreated patients.

**Conclusions:**

A high HOXB13 expression was associated with decreased benefit from tamoxifen, which indicates that HOXB13 protein level may be used as a predictive marker for tamoxifen treatment.

## Introduction

There have been several recent studies aimed at discovering novel biomarkers and gene signatures usable for predicting risk of recurrence and response to endocrine therapy of breast cancer [1-4]. With the development of robust, reliable genetic markers for this purpose, it would be possible at an early stage to predict which patients would benefit from alternative hormonal therapies. Resulting gene signatures, based on genome-wide microarray analyses, are often very comprehensive and comprise a large number of genes. However, Ma and colleagues were able to show that their gene expression profiles of hormone receptor-positive invasive breast tumors could be reduced into a simple two-gene ratio predictive of tumor relapse in the setting of adjuvant tamoxifen monotherapy [5]. Subsequent studies of the *HOXB13:IL17BR *index have proven its significance in predicting risk of breast cancer recurrence and tamoxifen response [6-9].

Our previous study of the *HOXB13:IL17BR *expression ratio indicated that the two genes individually could function as separate prognostic and treatment predictive markers in breast cancer [8]. Expression of *IL17BR *was inversely correlated to a number of factors related to a poor prognosis, whereas *HOXB13 *could predict recurrence in tamoxifen-treated patients. Patients with tumors expressing a high level of *HOXB13 *were more likely to be unresponsive to the therapy, suggesting that this gene is involved in tamoxifen resistance.

*HOXB13 *is a member of the homeobox gene family, a group of genes encoding transcriptional regulators of cell growth and differentiation, predominantly during embryogenesis. Much is known about the function of the homeobox genes in these events, but the role of *HOXB13 *in breast cancer and endocrine resistance is only beginning to be elucidated. The expression of *HOXB13 *is known to be upregulated in breast cancer cells compared with normal breast epithelium [5,10] and it has also been shown that *HOXB13 *is an estrogen-regulated gene negatively correlated to estrogen receptor (ER) status [11,12]. Wang and colleagues suggest that a high *HOXB13:IL17BR *index may indicate impaired ER signaling, which is known to predict resistance to tamoxifen [12].

To our knowledge, there are no studies investigating the HOXB13 protein levels in breast cancer and its significance in predicting outcome after tamoxifen treatment. In the present study, we used immunohistochemistry to analyze the protein expression of HOXB13 in tumor samples from 912 postmenopausal breast cancer patients. The patients were participants in a randomized trial analyzing the benefit from adjuvant treatment with tamoxifen, which enabled us to investigate the treatment predictive value of HOXB13.

## Materials and methods

### Patients

We analyzed tumor tissue from patients enrolled in a randomized tamoxifen trial comprising a total of 1,780 low-risk breast cancer patients, conducted in the Stockholm region in Sweden from 1976 to 1990 [13]. All patients were postmenopausal at the time of diagnosis, presented a tumor size ≤30 mm, and displayed no nodal involvement (N0). The patients were randomized to 2 years of endocrine treatment with tamoxifen (40 mg daily) or no adjuvant endocrine treatment. In 1983 a new trial was initiated; recurrence-free patients were, after 2 years of tamoxifen treatment, randomized to 3 years more of tamoxifen or no further therapy.

Tumor samples of 912 women from the trial were available for the present study, and tumor and treatment characteristics of the patients are presented in Table 1. The clinicopathological characteristics in this subset were similar to those in the complete series of 1,780 patients in the trial, such as tumor size ≤20 mm (79% vs. 81%), positive ER status (78% vs. 80%), and tamoxifen treatment (52% vs. 50%). The standard procedure for tissue collection was fixation in 4% phosphate-buffered formalin. Follow-up data were collected from regional population registers and the Swedish Cause of Death Registry. The mean follow-up period for patients in the present investigation was 17 years.

**Table 1:** Patient characteristics

	Patients in present study (*n *= 912)	Patients with HOXB13 expression data (*n *= 866)	Original cohort (*n *= 1,780)
Tumor diameter			
≤20 mm	697 (79)	661 (79)	1,393 (81)
>20 mm	189 (21)	181 (21)	323 (19)
Unavailable	26	24	64
Estrogen receptor status			
Positive	686 (78)	651 (77)	1,183 (80)
Negative	198 (22)	190 (23)	296 (20)
Unavailable	28	25	301
Progesterone receptor status			
Positive	415 (52)	401 (52)	590 (48)
Negative	380 (48)	366 (48)	627 (52)
Unavailable	117	99	563
Tamoxifen treatment			
Yes	473 (52)	447 (52)	886 (50)
No	439 (48)	419 (48)	894 (50)

Data presented as number of patients (%). Tumor and treatment characteristics of the patients included in the original cohort, patients in the present study and patients in the present study with successful HOXB13 protein expression scoring.

The study was approved by the local ethical committee at the Karolinska University Hospital. According to the approval, informed consent from the patients was not required.

### Hormone receptor status

The status of the ER and the progesterone receptor (PR) was assessed retrospectively with immunohistochemistry using the Ventana^® ^automated slide stainer (Ventana Medical Systems, S.A., Illkirch, France). Primary monoclonal antibodies were the CONFIRM™ mouse anti-ER antibody (clone 6F11) and the CONFIRM™ mouse anti-PR antibody (clone 16) from Ventana Medical Systems. The cut-off level was set to 10% positively stained tumor cell nuclei. In cases where immunohistochemical data for ER were missing (13%), the ER status determined in clinical routine practice [14] was used with a cut-off level of 0.10 fmol/μg DNA.

### Tissue microarray and immunostaining of HOXB13

Formalin-fixed and paraffin-embedded tissue blocks were selected as donor blocks for tissue microarray. Sections were cut from each donor block and were stained with hematoxylin and eosin. Three morphologically representative regions were chosen in each of the 912 tumor samples. Triplicate core tissue samples with a diameter of 0.8 mm were taken from selected areas of the tumors with a manual arrayer instrument (Beecher Instruments, Inc, Sun Prairie, WI, USA), and were arrayed into a paraffin block. Samples from human liver were included on each tissue block for orientation purpose.

Sections (4 μm thick) from the tissue microarray blocks were cut and mounted onto frost-coated glass slides, deparaffinized in xylene and rehydrated through a graded alcohol series to distilled water. The slides were boiled in 10 mM citrate buffer with pH 6.0, in a pressure cooker for 3 minutes. After cooling to room temperature, the slides were washed and incubated for 5 minutes in 3% hydrogen peroxide and methanol, and were then incubated with serum-free protein block (Dako Sweden AB, Stockholm, Sweden). A mouse monoclonal antibody raised against amino acids 1 to 284 of human HOXB13 (F-9 sc-28333, lot number L1504; Santa Cruz Biotechnology, Inc., Heidelberg, Germany) was applied as primary antibody at a 1:50 dilution, and the slides were incubated at 4°C for 16 hours. As the secondary reagent, the EnVision™ + Dual Link System labeled with horseradish peroxidase (Dako Sweden AB) for 30 min at room temperature was used. The slides were incubated with 3,3'-diaminobenzidine tetrahydrochloride solution, counterstained with hematoxylin, and mounted. BT-474 cells were used as a positive control. All washing steps were performed in phosphate-buffered saline with 0.5% bovine serum albumin.

### Evaluation of immunohistochemical staining

The stained tumor sections were evaluated independently by two investigators (P-LJ and OS) without knowledge about the clinicopathological data. The slides were examined using a Leica LB30T microscope (Leica Microsystems, Wetzlar, Germany) and were photographed using an Olympus SC20 digital camera (Olympus Europe GmbH, Hamburg, Germany). Nuclear staining intensity was graded as negative, weak, moderate or strong. In cases with different scoring results, a consensus score was reached after re-evaluation. In the survival analysis, weak or no staining was categorized as low expression and moderate to strong staining was considered high expression.

### Immunohistochemical analysis of HER2

Formalin-fixed, paraffin-embedded tissue was stained for human epidermal growth factor receptor 2 (HER2) with the DAKO AO0485 polyclonal rabbit antibody according to the guidelines provided by the manufacturer. The slides were scored as follows: 0 = no staining or <10% of the cells positive for membrane staining; 1 = weak or barely perceptible staining in >10% of the cells, staining in only part of the cell membrane; 2 = weak to moderate staining in the whole membrane in >10% of the cells; and 3 = strong staining in the whole membrane displayed by >10% of the cells. In all cases, the scoring was limited to the invasive tumor.

### Western blot

Lysates from the cell lines BT-474, SKBR3 and T47 D were used to determine the specificity of the HOXB13 antibody. Real-time PCR analysis of *HOXB13 *mRNA shows that SKBR3 and T47 D express very low, if any, levels of *HOXB13*, whereas BT-474 cells show high expression (unpublished data). The lysates were loaded on a 4 to 15% gradient precast gel (Criterion; Bio-Rad Laboratories AB, Sundbyberg, Sweden) and the proteins were then transferred to a polyvinylidene fluoride membrane. The membrane was incubated overnight with the antibody (1:750) or with antibody previously incubated with full-length recombinant HOXB13 protein in a 10-fold excess. Antibodies were detected with a commercial Enhanced Chemiluminescence Plus kit (GE Healthcare UK Ltd, Little Chalfont, UK). To control for equal loading, the membrane was stripped and then incubated with a rabbit monoclonal GAPDH antibody (1:1,000; Cell Signaling Technology, Inc., Danvers, MA, USA) and a secondary polyclonal goat-anti-rabbit antibody (1:2,000; Dako).

### Statistical analysis

Survival curves were produced according to the life-table method described by Kaplan and Meier, and differences in survival were estimated with the log-rank test. Distant recurrence-free survival (DRFS) was defined as the time from diagnosis to distant recurrence or to death due to breast cancer. Breast cancer survival (BCS) was the time elapsed from diagnosis to the date of death related to breast cancer.

*P *values for relationships between HOXB13 and other variables were assessed with a chi-square test for trend. Univariate analyses of recurrence rates were performed with Cox proportional hazard regression. Tests of interactions between treatment effect and HOXB13 analysis were performed by including product terms in the models, as well as other prognostic factors (tumor size and HER2). All statistical procedures are comprised in the statistical package Statistica 9.1 (StatSoft Scandinavia AB, Uppsala, Sweden).

## Results

### HOXB13 protein expression

**Figure 1:**
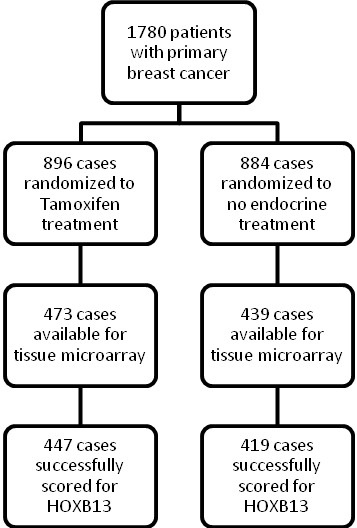
**Study design**. Randomization and flow of patients included in the original study and in the present study.

**Figure 2:**
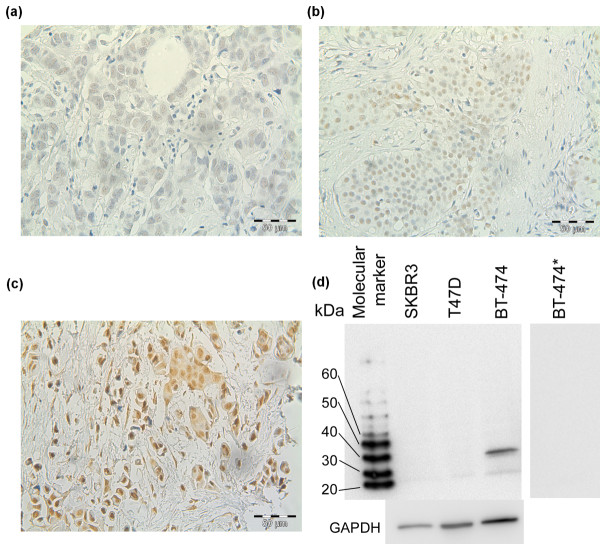
**HOXB13 grading and antibody control**. Tumor tissue immunostained for HOXB13: **(a) **weak, **(b) **moderate and **(c) **strong. **(d) **Western blot analysis of HOXB13 in protein lysates from SKBR3 cells, T47 D cells and BT-474 cells. Lane BT-474*, antibody was preincubated with recombinant HOXB13 protein prior to incubation with the membrane.

Immunohistochemical staining of HOXB13 was performed on tissue microarray slides comprising a total of 912 tumors from breast cancer patients. The flow of patients through the study is described in Figure 1. Grading of the protein expression was successful in 866 cases (95% of the total number of patients), of which 291 (33.6%) displayed negative staining, 317 (36.6%) weak staining, 212 (24.4%) moderate staining, and 46 (5.3%) strong staining. Expression of HOXB13 was located exclusively in the nuclei of the tumor cells. Examples of the different levels of immunostaining are shown in Figure 2a to 2c. Western blot analysis of the specificity of the antibody gave a single specific band at 34 kDa, which was not detectable when the antibody was pre-incubated with recombinant HOXB13 protein (Figure 2d).

### Expression of HOXB13 in relation to prognosis and prediction of treatment outcome

Among the patients included in the present study, there were 419 patients with HOXB13 protein expression data who did not receive any adjuvant endocrine treatment - 74% of which had ER-positive disease (*n *= 310, 16 patients had unknown ER status). For all of the endocrine-untreated patients, irrespective of ER status, neither the rate of distant recurrence nor the breast cancer-related survival differed between patients with different levels of HOXB13 protein expression in the tumor. Hazard ratios according to univariate Cox proportional hazard modeling for patients presenting high expression compared with patients presenting low expression were not statistically significant (DRFS hazard ratio = 0.89, 95% confidence interval = 0.58 to 1.36, *P *= 0.59; BCS hazard ratio = 0.76, 95% confidence interval = 0.46 to 1.25, *P *= 0.28). Restricting the analysis to the ER-positive group gave similar results (DRFS hazard ratio = 0.78, 95% confidence interval = 0.47 to 1.29, *P *= 0.32; BCS hazard ratio = 0.60, 95% confidence interval = 0.32 to 1.14, *P *= 0.11).

**Figure 3:**
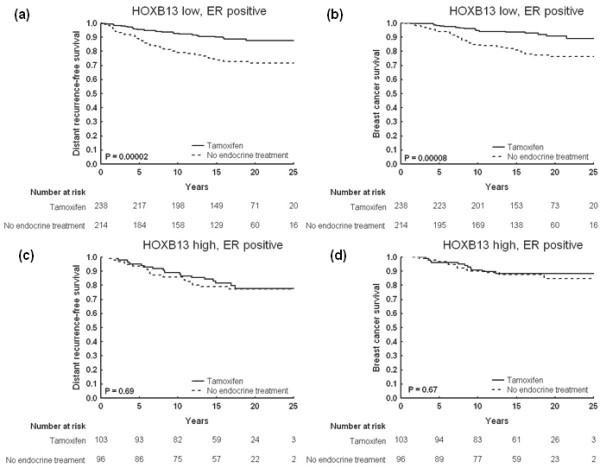
**Cumulative survival for patients with estrogen receptor-positive tumors, stratified by HOXB13**. Kaplan-Meier curves illustrating clinical outcome in terms of distant recurrence-free survival and breast cancer survival for patients with estrogen receptor (ER)-positive tumors undergoing tamoxifen treatment or no endocrine therapy: **(a), (b) **low or no HOXB13 expression; **(c), (d) **moderate or high HOXB13 expression.

Tamoxifen significantly increased the DRFS (*P *= 0.00002) and the BCS (*P *= 0.00008) in patients with ER-positive tumors expressing none or low levels of HOXB13 (Figure 3a,b), whereas the patients with a moderate to high HOXB13 expression in their tumors did not benefit from the endocrine treatment (Figure 3c,d). The interaction between HOXB13 expression and treatment effect was statistically significant for DRFS (*P *= 0.035; multivariate model adjusted for tumor size and HER2 status). Cox proportional hazard regressions for the tamoxifen benefit in the different patient groups are shown in Table 2.

**Table 2:** Estimation of the benefit from adjuvant tamoxifen treatment

Tumor status	Tamoxifen treatment versus no endocrine treatment					
	
	Distant recurrence			Breast cancer death		
		
	Hazard ratio (95% CI)	*P *value	*P*_interaction_*	Hazard ratio (95% CI)	*P *value	*P*_interaction_*
ER-positive						
Low HOXB13 expression	0.38 (0.23 to 0.60)	0.000048		0.35 (0.20 to 0.60)	0.00016	
High HOXB13 expression	0.88 (0.47 to 1.65)	0.69	0.035	0.84 (0.37 to 1.90)	0.67	0.060
ER-positive, PR-positive						
Low HOXB13 expression	0.26 (0.14 to 0.49)	0.000022		0.24 (0.11 to 0.50)	0.00015	
High HOXB13 expression	0.70 (0.31 to 1.56)	0.38	0.072	0.82 (0.27 to 2.56)	0.74	0.059
ER-positive, PR-negative						
Low HOXB13 expression	0.69 (0.31 to 1.56)	0.37		0.64 (0.26 to 1.56)	0.33	
High HOXB13 expression	0.89 (0.31 to 2.54)	0.83	0.51	0.60 (0.17 to 2.13)	0.43	0.98

Cox regression analysis of distant recurrence rate and breast cancer-related deaths for patients with estrogen receptor (ER)-positive tumors, ER-positive and progesterone receptor (PR)-positive tumors, and ER-positive and PR-negative tumors in relation to HOXB13 protein expression. CI, confidence interval. *Tests whether there is a difference in the treatment response. The models included treatment (tamoxifen vs. no endocrine treatment), HOXB13 expression (high vs. low), an interaction variable, tumor size (>20 mm vs. ≤20 mm) and HER2 status (3+ vs. 0 to 2+).

**Figure 4:**
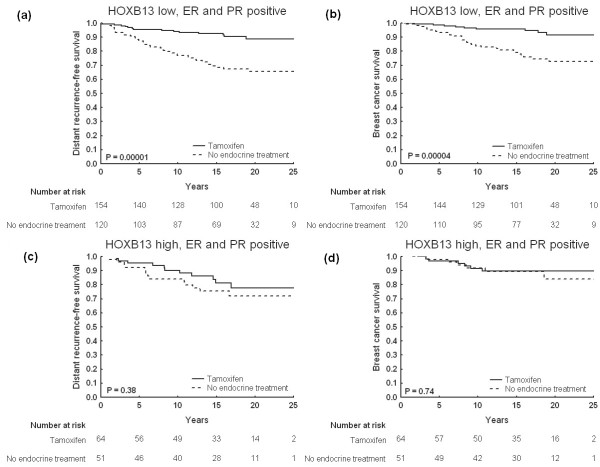
**Cumulative survival for patients with tumors expressing estrogen receptors and progesterone receptors, stratified by HOXB13**. Kaplan-Meier curves illustrating clinical outcome in terms of distant recurrence-free survival and breast cancer survival for patients with estrogen receptor (ER)-positive and progesterone receptor (PR)-positive tumors undergoing tamoxifen treatment or no endocrine therapy: **(a), (b) **low or no HOXB13 expression; **(c), (d) **moderate or high HOXB13 expression.

For patients with tumors positive for both ER and PR, survival analysis indicated that there was a significant benefit from tamoxifen if the HOXB13 expression was low (DRFS, *P *= 0.00001; BCS, *P *= 0.00004) (Figure 4a,b). High levels of HOXB13 were still unfavorable for the patients; no difference in relapse-free survival was noted comparing the treated and untreated groups of patients (DRFS, *P *= 0.38; BCS, *P *= 0.74) (Figure 4c,d). *P *values for the multivariate interaction analyses, however, did not reach statistical significance (*P *= 0.072 and *P *= 0.059 for DRFS and BCS, respectively; Table 2). Further analyses showed that patients with ER-positive but PR-negative tumors did not seem to benefit from tamoxifen, regardless of HOXB13 expression levels (Table 2).

### Correlation with prognostic factors

Analysis of possible relationships between HOXB13 protein expression and different tumor variables revealed correlations to HER2, ER and tumor size (Table 3). HOXB13 was inversely correlated to tumor size (*P *= 0.0048) and positively correlated to ER (*P *= 0.0022) and HER2 levels (*P *= 0.0023).

**Table 3:** Correlation between HOXB13 protein levels and prognostic factors analyzed with the chi-square test for trend

	HOXB13 expression				*P *value
		
	Negative	Low	Intermediate	High	
Tumor size					
≤20 mm	205 (31.0)	249 (37.7)	168 (25.4)	39 (5.9)	
>20 mm	78 (43.1)	59 (32.6)	37 (20.4)	7 (3.9	0.0048
Human epidermal growth factor receptor 2					
0	189 (38.4)	172 (35.0)	102 (20.7)	29 (5.9)	
1+	38 (26.6)	55 (38.5)	45 (31.5)	5 (3.5)	
2+	16 (20.5)	32 (41.0)	26 (33.3)	4 (5.1)	
3+	27 (29.7)	30 (33.0)	27 (29.7)	7 (7.7)	0.0023
Estrogen receptor*					
0	63 (39.4)	48 (30.0)	34 (21.3)	15 (9.4)	
1 to 24	5 (35.7)	5 (35.7)	4 (28.6)	0 (0)	
25 to 74	69 (57.0)	35 (28.9)	16 (13.2)	1 (0.8)	
75 to 89	63 (33.3)	67 (35.5)	47 (24.9)	12 (6.4)	
>90	62 (22.1)	110 (39.3)	96 (34.3)	12 (4.3)	0.0022
Progesterone receptor*					
0	120 (39.9)	92 (30.6)	68 (22.6)	21 (7.0)	
1 to 24	43 (42.6)	29 (28.7)	26 (25.7)	3 (3.0)	
25 to 74	58 (29.3)	87 (43.9)	48 (24.2)	5 (2.5)	
75 to 89	27 (24.1)	42 (37.5)	33 (29.5)	10 (8.9)	
>90	20 (36.4)	21 (38.2)	12 (21.8)	2 (3.6)	0.13

Data presented as number of patients (%).*Percentage of positive nuclei stained with immunohistochemistry.

## Discussion

The results from studies aiming to find molecular markers to predict treatment response in breast cancer demonstrate the importance of sensitive markers for optimizing and individualizing breast cancer therapy [1-4]. Several studies have reported an association between *HOXB13 *mRNA expression and clinical outcome [5-9,12]. In the present study, 34% of the tumors were negative for HOXB13 protein expression - which correlates well to the proportion of tumors with undetectable mRNA expression in the study by Jansen and colleagues [7].

The present report is the first about HOXB13 protein expression and its potential as a predictor of response to endocrine treatment. In the systemically untreated group, HOXB13 could not predict the clinical outcome. This is in line with our previous findings, examining potential prognostic features of mRNA expression [8]. The clinical value of HOXB13 seems to be within its predictive power. As noted, a high expression of the protein results in less benefit from endocrine treatment with tamoxifen. Most of the previous studies of *HOXB13 *have included mainly lymph node-negative patients, but we have previously shown that the predictive feature of the two-gene ratio *HOXB13:IL17BR *is also applicable to patients with nodal involvement [8]. Since the present study comprises a cohort of only lymph node-negative patients, it would be interesting to examine the HOXB13 protein expression in patients with nodal involvement.

The causes of a high HOXB13 expression in breast tumors are still not completely clear. The gene encoding HOXB13 maps to chromosome 17q21, a region known to be amplified in breast cancer. The gene encoding HER2 also maps to this chromosomal location, which raises the question about a possible coamplification of the two genes. This is one potential reason for the positive correlation between the protein expressions we have seen in the present study, a result that is in line with a previous study by Wang and colleagues [12]. In that study, however, *HOXB13 *displayed an ER-dependent correlation with HER2 status, since the correlation was evident only in ER-positive tumors. In our study, this relationship was evident in both ER-positive tumors as well as ER-negative tumors.

The correlation analysis of HOXB13 protein expression and other proteins in the present study revealed a positive correlation to ER; however, mRNA levels of *HOXB13 *have been reported to correlate negatively with ER [7,12]. There is also evidence pointing towards *HOXB13 *being an ER-responsive gene, with estradiol treatment resulting in a suppression of the gene [11,12]. Given that the intrinsic levels of estradiol are highly variable between different breast tumors [15,16] and that HOXB13 may be a coactivating factor in ER signaling [17], one may speculate that high HOXB13 protein levels can contribute to increased ER activity despite a low estradiol concentration within the tumor. A decreased estradiol concentration can result from a lower intrinsic production, or can occur during treatment with aromatase inhibitors. In fact, it seems HOXB13 is not only a predictor of tamoxifen resistance, but is a marker of resistance to endocrine therapy *per se*. In a recent study by Ma and colleagues, *HOXB13 *gene expression was shown to predict benefit from treatment with the aromatase inhibitor letrozole [18]. In the future, a study including both HOXB13, ER and estradiol concentrations in a set of tumors would be welcome.

The mechanistic effects of HOXB13 in breast cancer are being investigated, but there are few studies published in this matter. The two-gene ratio *HOXB13*:*IL17BR *has been proposed as a marker for impaired ER gene regulation [12]. The presence of PR in a tumor is the most commonly used indicator of functional ER signaling [19]; patients with tumors expressing both ER and PR benefit most from tamoxifen therapy. The worse prognosis when HOXB13 is high, which is seen in our study, shows that HOXB13 in fact has a predictive value in addition to PR.

## Conclusions

The present study is the first about HOXB13 protein expression in breast cancer. We have shown that HOXB13 is predictive of response to adjuvant tamoxifen therapy, in terms of a longer DRFS and BCS for tamoxifen-treated breast cancer patients if the protein expression is low or absent. Nevertheless, further studies are needed to verify our results.

## Abbreviations

BCS: breast cancer survival; DRFS: distant recurrence-free survival; ER: estrogen receptor; HER2: human epidermal growth factor receptor 2; PCR: polymerase chain reaction; PR: progesterone receptor.

## Competing interests

The authors declare that they have no competing interests.

## Authors' contributions

P-LJ participated in the study design, performed the laboratory work, evaluated the immunohistochemical staining, performed the statistical analyses, and wrote the manuscript. AJ participated in the study design, performed laboratory work, and provided critical revision of the manuscript. TF, LS, and BN provided the study material and clinical information, and collected laboratory data on the study patients. OS participated in the study design, evaluated the immunohistochemical staining, performed the statistical analyses, and provided critical revision of the manuscript. All authors read and approved the final manuscript.
